# The fusion of multiple scale data indicates that the carbon sink function of the Qinghai-Tibet Plateau is substantial

**DOI:** 10.1186/s13021-023-00239-9

**Published:** 2023-09-11

**Authors:** Jingyu Zeng, Tao Zhou, Yixin Xu, Qiaoyu Lin, E. Tan, Yajie Zhang, Xuemei Wu, Jingzhou Zhang, Xia Liu

**Affiliations:** 1 Key Laboratory of Environmental Change and Natural Disasters of Chinese Ministry of Education, Beijing Normal University, Beijing, 100875 China; 2 State Key Laboratory of Earth Surface Processes and Resource Ecology (ESPRE), Beijing Normal University, Beijing, 100875 China

**Keywords:** Qinghai-Tibet Plateau, Carbon source and sink, Multiple scale information fusion, Machine learning, Uncertainty assessment

## Abstract

**Background:**

The Qinghai-Tibet Plateau is the “sensitive area” of climate change, and also the “driver” and “amplifier” of global change. The response and feedback of its carbon dynamics to climate change will significantly affect the content of greenhouse gases in the atmosphere. However, due to the unique geographical environment characteristics of the Qinghai-Tibet Plateau, there is still much controversy about its carbon source and sink estimation results. This study designed a new algorithm based on machine learning to improve the accuracy of carbon source and sink estimation by integrating multiple scale carbon input (net primary productivity, NPP) and output (soil heterotrophic respiration, Rh) information from remote sensing and ground observations. Then, we compared spatial patterns of NPP and Rh derived from the fusion of multiple scale data with other widely used products and tried to quantify the differences and uncertainties of carbon sink simulation at a regional scale.

**Results:**

Our results indicate that although global warming has potentially increased the Rh of the Qinghai-Tibet Plateau, it will also increase its NPP, and its current performance is a net carbon sink area (carbon sink amount is 22.3 Tg C/year). Comparative analysis with other data products shows that CASA, GLOPEM, and MODIS products based on remote sensing underestimate the carbon input of the Qinghai-Tibet Plateau (30–70%), which is the main reason for the severe underestimation of the carbon sink level of the Qinghai-Tibet Plateau (even considered as a carbon source).

**Conclusions:**

The estimation of the carbon sink in the Qinghai-Tibet Plateau is of great significance for ensuring its ecological barrier function. It can deepen the community’s understanding of the response to climate change in sensitive areas of the plateau. This study can provide an essential basis for assessing the uncertainty of carbon sources and sinks in the Qinghai-Tibet Plateau, and also provide a scientific reference for helping China achieve “carbon neutrality” by 2060.

**Supplementary Information:**

The online version contains supplementary material available at 10.1186/s13021-023-00239-9.

## Highlights

1. This study designed a carbon sink estimation method for Qinghai-Tibet Plateau by integrating machine learning and multiple scale ground- and remote sensed-based data.
2. The estimated total carbon sink of the Qinghai-Tibet Plateau is 22.3 Tg C/year, accounting for about 10% of China's total carbon sink.
3. The carbon sink of former estimation maybe greatly underestimated due to the underestimation of carbon input in the Qinghai-Tibet Plateau.

**Supplementary Information:**

The online version contains supplementary material available at 10.1186/s13021-023-00239-9.

## Background

Terrestrial ecosystems have always been the largest natural carbon sink. Especially since the 1960s, more than a quarter of anthropogenic emissions have been stored on average [1]. Considering its importance in combating climate warming, accurate estimation of carbon sinks is crucial for assessing mitigation policies [2–4]. With the development of technology, significant progress has been made in the annual updated global carbon estimate [1, 5]. However, in terms of its response, such as the trend of atmospheric CO_2_ and the increase of interannual variability, climate variability and other environmental factors, the terrestrial carbon sink is still highly uncertain [6–10]. These uncertainties are the main obstacle to our ability to predict climate feedback and its potential impact on the human-natural system [11].

As a unique geomorphic unit on the earth, the Qinghai-Tibet Plateau (QTP) is very sensitive to global warming [12], and its warming rate in the past 50 years is twice the global average [13–15]. It is observed that the permafrost on the Qinghai-Tibet Plateau has been significantly degraded due to the rapid warming of the climate [12, 16]. These changes have a significant impact on the ecology, hydrology, biogeochemistry and engineering infrastructure of the third pole and surrounding areas [17, 18]. In particular, the melting of permafrost will cause the release of soil organic carbon (SOC) into the atmosphere, which will affect climate warming [19–21]. At the same time, the Qinghai-Tibet Plateau is considered to have a profound impact on the regional and global climate system and ecological economy [22]. It is a sensitive indicator of climate change and a key component of the global carbon (C) cycle [23]. The response of the Qinghai-Tibet Plateau C dynamics to climate change may significantly affect the concentrations of carbon dioxide (CO_2_) and methane (CH_4_) in the atmosphere [24, 25], thus affecting the pace of future climate change [26, 27].

At present, whether the Qinghai-Tibet Plateau is a carbon source or a carbon sink is controversial [28]. For example, based on ORCHIDE and TEM models, the Qinghai-Tibet Plateau (QTP) is estimated to be a carbon sink [29, 30]. The results of CASA model and empirical statistical model show that the grassland ecosystem in the central and southern parts of the Qinghai-Tibet Plateau shows weak net carbon absorption, and its net ecosystem productivity (NEP) is 42.03 g C/m^2^ year [31]. The research based on the traditional empirical model believes that the Qinghai-Tibet Plateau is a carbon source [32, 33]. Studies also found that the Qinghai-Tibet Plateau is a carbon sink at present, but in the case of climate warming at the end of the twenty-first century, it will change from carbon sink to carbon source [34]. Koven et al. [35] revealed that by the end of the twenty-first century, the climate was warming, and the permafrost ecosystem in high latitudes could shift from carbon sink to carbon source. Although the warming of the Qinghai-Tibet Plateau will increase the carbon sink capacity of the ecosystem in the growing season, it will also increase the soil carbon decomposition in the non-growing season, and the degradation of the permafrost region will increase the carbon release of the ecosystem. This will also destroy the surface vegetation, causing the ecosystem to change from carbon sink to carbon source in the growing season [12]. However, due to the high spatiotemporal heterogeneity of the Qinghai-Tibet Plateau, it is challenging to simulate, evaluate and predict the spatiotemporal dynamics of carbon flux [15, 36].

The significant uncertainty in estimating carbon sinks in the Qinghai Tibet Plateau is due to differences in methods. The estimation methods for carbon sources and sinks in terrestrial ecosystems include “top-down” atmospheric inversion method, “bottom-up” inventory method, vorticity correlation method, and ecosystem process model simulation method [19, 37, 38]. These methods may be limited to varying degrees by model parameters, number and distribution of stations, scale conversion and simulation accuracy, especially in the Qinghai Tibet Plateau region, where the differences in estimation results between different methods may exceed 10 times [12, 39]. Due to the complex underlying surface conditions and high spatial heterogeneity of the Qinghai Tibet Plateau, it is difficult to localize the parameters of the process model [38]. Remote sensing methods are also not suitable for most areas of the sparsely vegetated Qinghai Tibet Plateau. The carbon source and sink estimation method that combines remote sensing products with ground observation data to ensure spatial continuity and quantitative accuracy is very promising [40, 41]. This data-driven method can retain the effective information of remote sensing products and sample observation data, capture the complex nonlinear relationship between input and output variables, and achieve the goal of unifying different data scales, so it has high flexibility and data adaptability [42, 43]. Based on the data-driven method, new information can also be mined from data to promote a new understanding of the new mechanism [44]. Therefore, the multiple scale information fusion method of carbon sink estimation using machine learning as a bridge to connect multiple-source data such as remote sensing and ground sample observation data is an effective solution to reduce the uncertainty of the results.

In order to more accurately simulate the size and spatial pattern of carbon sources and sinks in the Qinghai-Tibet Plateau, we designed a multiple scale information fusion method for carbon sink estimation that uses machine learning as a bridge to connect multiple-source data such as remote sensing and ground sample observation data. Thus, a more accurate and spatially continuous carbon source and sink and its spatial pattern from 2000 to 2018, which can reflect the regional characteristics of the Qinghai-Tibet Plateau, are obtained. Then, we compared the ecosystem carbon input and carbon output with other widely used similar products, and discussed the differences and uncertainties of carbon sink simulation in the Qinghai-Tibet Plateau.

## Data sources and methods

### Study area

**Fig. 1:**
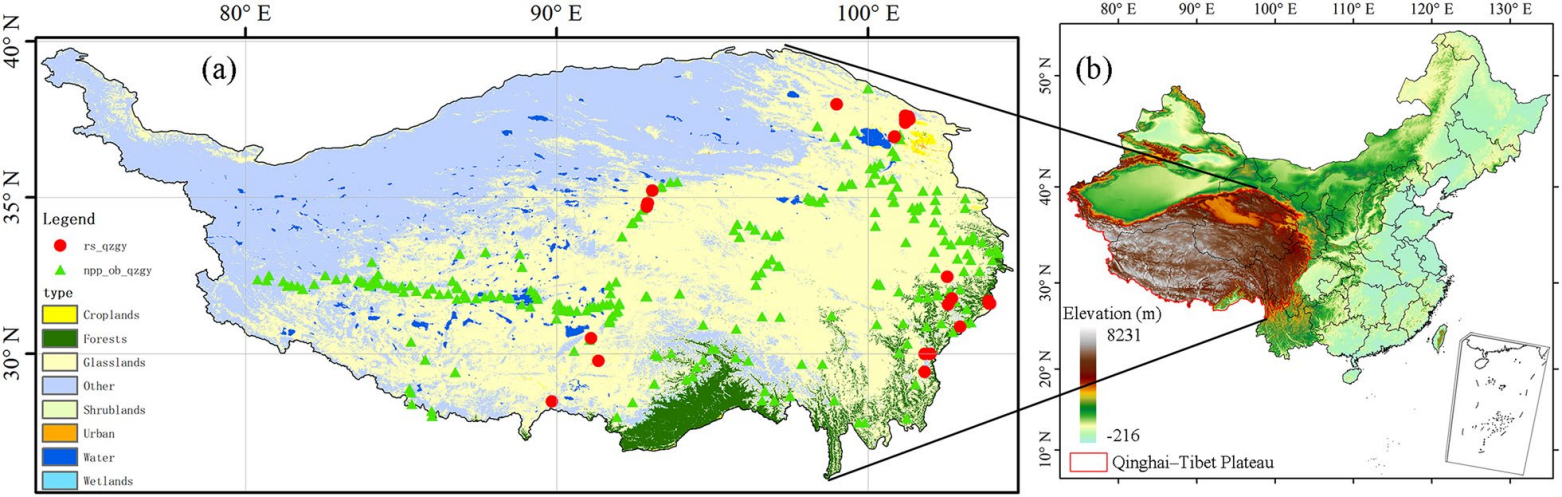
Overview of the study area. **a** Land use types and location of site observations of ground sample in the Qinghai-Tibet Plateau; **b** The elevation of the Qinghai-Tibet Plateau and its position in China

The Qinghai-Tibet Plateau locates in western China (73.50–104.67° E, 25.99–39.83° N), with an area of more than 2.56 × 10^6^ km^2^ (Fig. 1). As an Asian water tower, about 40% of the world's population depends on or is affected by it [45]. Grassland is the main vegetation type in the Qinghai-Tibet Plateau. Since the twenty-first century, the annual average temperature in the coldest region of the Qinghai-Tibet Plateau has been lower than − 20 °C, while that in the warmest region is higher than 20 °C [46]. Since the mid-1950s, the Qinghai-Tibet Plateau has experienced significant warming, with the annual average temperature increasing by 0.3 °C every decade [30]. The spatial heterogeneity of precipitation on the Qinghai-Tibet Plateau is extremely high. The southeast Qinghai-Tibet Plateau is the wettest region with an annual precipitation of more than 1000 mm. The annual precipitation in the driest area in the northwest is less than 50 mm [47].

### Data sources

#### Ground observation data

NPP ground observation data are from relevant literature, and soil respiration and heterotrophic respiration ground observation data are from the fifth edition of soil respiration database [48]. Ground observation data are used for model construction and precision evaluation. The details for these ground observation list in appendix.

#### Spatial data

Three NPP product data sets were used in this study. MOD17A3H has a spatial resolution of 500 m, a time resolution of years and a time span of 2000–2018(NPP_MODIS_). Chen [49] estimated China’s 1 km NPP product (NPP_CASA_) from 1985 to 2015 based on CASA model. Institute of Geographic Sciences and Natural Resources, Chinese Academy of Sciences based on light energy utilization model GLO_PEM estimated China’s 1 km NPP product (NPP_GLO_PEM_) from 2000 to 2010. The random forest model uses the high-resolution meteorological driven data set developed by He et al. [50] for input, including air temperature, rainfall and downward short-wave radiation. The spatial resolution is 0.1°, the temporal resolution is years, and the time span is 1979–2018. The product data of land cover type includes MCD12Q1, with a spatial resolution of 500 m, a temporal resolution of years, and a time span of 2001–2018. The leaf area index (LAI) data is from Liu et al. [51], with a spatial resolution of 8 km and a temporal resolution of 8d, and a time span of 1981–2019. Digital Elevation Model (DEM) includes SRTMDEMUTM products with a spatial resolution of 90 m. The global soil organic carbon (SOC) comes from the soil geographic database with a spatial resolution of 250 m. The global 1 km spatial resolution soil respiration data (Rs_QRFM_) estimated by quantile regression forest model is from Stell et al. [52]. The above data were resampled by the nearest neighbor at a spatial resolution of 1 km, and the annual data were synthesized by the average method.

### Methods

#### NPP estimate

The random forest (RF) model includes many binary decision trees, which are grown independently using a two-stage randomization program [53]. Using a bootstrap resampling method, RF creates a tree structure classifier through independent and identically distributed random vectors, which randomly extracts quantitative samples as training samples. RF then constructs a decision tree model for each training sample set. The model obtains the final regression result by calculating the average value predicted by multiple decision trees [54]. A large number of theoretical and practical studies have proved that the RF algorithm has high accuracy and stability and has a good tolerance to outlier and noise [55]. Based on the NPP data observed on the ground, we built a random forest model by integrating the NPP data estimated by remote sensing and environmental variables, and simulated the NPP of China from 2000 to 2018. The model structure is as follows:

1 $$\mathrm{NPP}=\mathrm{f}(\mathrm{Precipitation},\mathrm{ Temperature},\mathrm{ SRAD},\mathrm{ LULC},\mathrm{ DEM},\mathrm{ LAI}, {\mathrm{NPP}}_{\mathrm{modis}})$$

where NPP is the net primary productivity of vegetation (g C/m^2^ year) after integrating ground observation and remote sensing information, SRAD is short-wave radiation, LULC is land cover type data, DEM is digital elevation model, LAI is leaf area index, NPP_modis_ is the NPP remote sensing product of MODIS, which has been widely used and has high spatial resolution [56, 57]. Using NPP_modis_ and LAI to replace the traditional vegetation index is a new idea in this study. It can effectively estimate the photosynthetic capacity and growth status of vegetation in complex environments [58, 59]. We estimate the relationship between NPP and independent variables through the random forest model established by R language. The ntree parameter in the model is set to 1500. Since there are seven input variables in the model, the mrty parameter is set to six.

#### Rs and Rh estimate

Considering that soil respiration (Rs) has more observation data than soil heterotrophic respiration (Rh), the estimation of Rh is carried out in two steps. First, soil respiration (Rs) is estimated by random forest model, and then Rh is further estimated by Rs. The random forest model of Rs is as follows:

2 $$\mathrm{Rs}=\mathrm{f}(\mathrm{Precipitation},\mathrm{ Temperature},\mathrm{SRAD},\mathrm{ LULC},\mathrm{ DEM},\mathrm{ LAI}, {\mathrm{NPP}}_{\mathrm{modis}},\mathrm{ SOC})$$

where Rs is soil respiration (g C/m^2^ year), SOC is soil organic carbon, and other variables are consistent with NPP model (Eq. 1). The parameter of ntree in the model is set to 1500. Since there are eight input variables in the model, the parameter of mrty is set to seven. After obtaining the simulated value of Chinese Rs, we further estimate the Rh of the Qinghai-Tibet Plateau based on the statistical model of Chinese Rs and Rh (Eq. 3) (see the Additional file 1: Fig. S1 for details).

3 $$\mathrm{Rh}=0.62*\mathrm{Rs}+9.01 {\mathrm{R}}^{2}=0.77$$

#### NEP estimate

Based on the estimated NPP and Rh, we calculate the net ecosystem productivity (NEP) of the Qinghai-Tibet Plateau:

4 $$\mathrm{NEP}=\mathrm{NPP}-\mathrm{Rh}$$

where NEP is the net productivity of the ecosystem (g C/m^2^yr), which is used to characterize the carbon source/sink level of the ecosystem. When NEP is positive, it indicates that the ecosystem is a carbon sink; When NEP is negative, it indicates that the ecosystem is a carbon source.

#### Evaluation indicators

Multiple indicators were used to evaluate the reliability of random forest models and the accuracy of different models. These indicators include decision coefficient, correlation coefficient (r), root mean square error (RMSE), percent bias (PBIAS), mean absolute percentage error (MAPE).

5 $$\mathrm{PBIAS}=\frac{\sum_{\mathrm{i}=1}^{\mathrm{n}}\left(\mathrm{pi}-\mathrm{Oi}\right)*100}{\sum_{\mathrm{i}=1}^{\mathrm{n}}\mathrm{Oi}}$$

6 $$\mathrm{RMSE}=\sqrt{\frac{\sum_{\mathrm{i}=1}^{\mathrm{n}}{\left(\mathrm{pi}-\mathrm{Oi}\right)}^{2}}{N}}$$

7 $${\text{MAPE}} = \frac{1}{n}\sum\nolimits_{t = 1}^n {\left| {\frac{{Oi - Pi}}{{Oi}}} \right|}$$

8 $$\mathrm{r}=\frac{\sum_{\mathrm{i}=1}^{\mathrm{n}}\left({\mathrm{x}}_{\mathrm{i}}-\overline{\mathrm{x} }\right)({\mathrm{y}}_{\mathrm{i}}-\overline{\mathrm{y} })}{\sqrt{\sum_{\mathrm{i}=1}^{\mathrm{n}}{\left({\mathrm{x}}_{\mathrm{i}}-\overline{\mathrm{x} }\right)}^{2}}\sqrt{\sum_{\mathrm{i}=1}^{\mathrm{n}}{\left({\mathrm{y}}_{\mathrm{i}}-\overline{\mathrm{y} }\right)}^{2}}}$$

where Pi is the i-th predicted value of the evaluated element, O_i_ is the i-th observed value, P is the average of the predicted values, O is the average of the observed values, and n is the total number of observations.

**Fig. 2:**
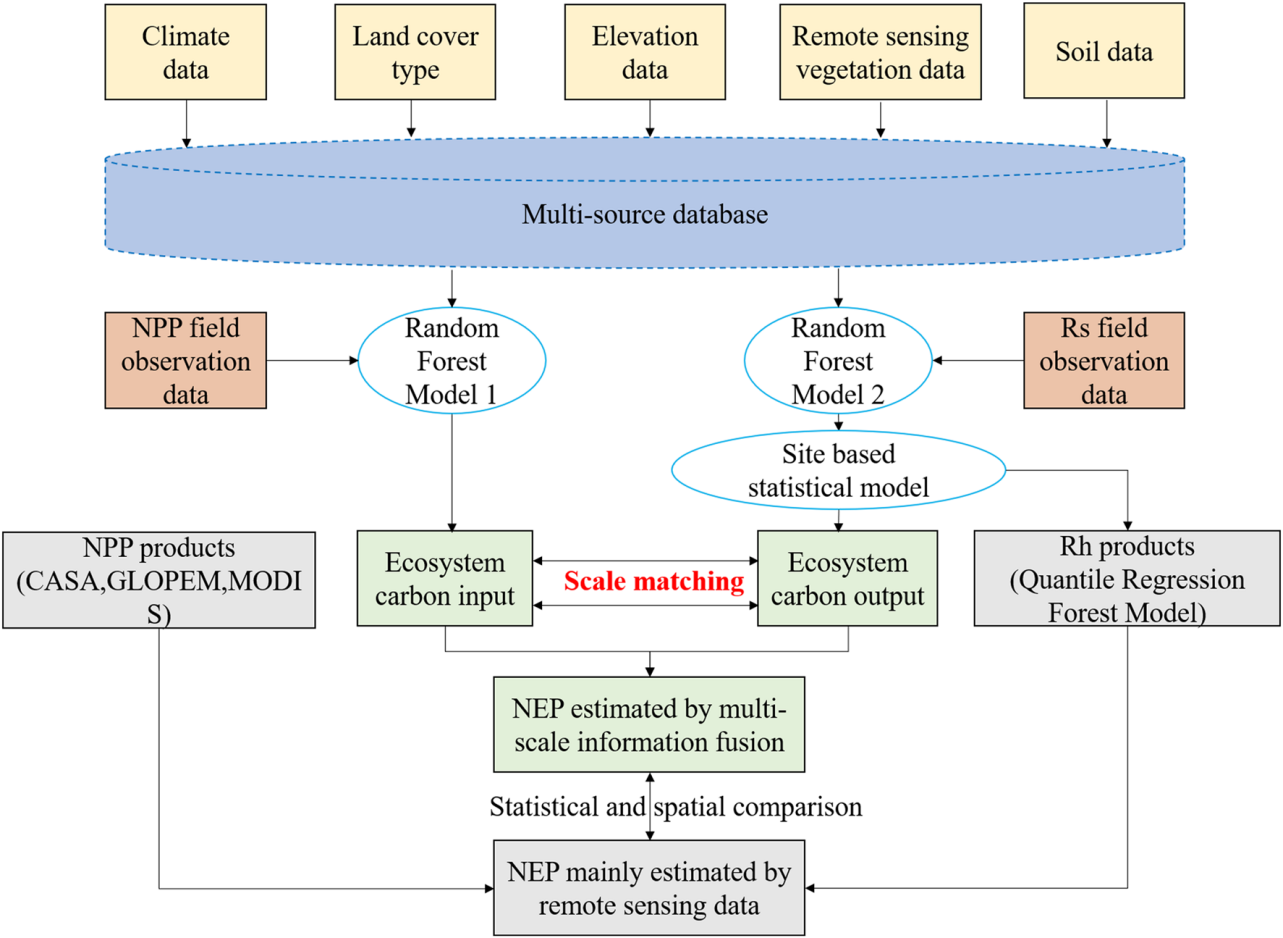
The flow framework of this study

The flow framework of this study is shown in Fig. 2. The idea of this study is to first build a database based on multi-source spatial and remote sensing data. Next, a random forest model is used as a bridge to connect ground sample observation data and spatial data to address the system bias caused by scale differences between different data. Simultaneously establish two sets of random forest models to simulate NPP and Rs, so that the scale of simulated carbon input and carbon output data matches. Finally, multiple indicators are used to evaluate the simulation accuracy of different models.

## Results

### NPP estimation of Qinghai-Tibet Plateau

The carbon flux estimation model of NPP and Rs is constructed by using the random forest model. The simulation results of the model are reliable and accurate (Additional file 1: Figs. S2 and S3). The R^2^ value of the random forest model based on 394 NPP ground samples in the Qinghai-Tibet Plateau is 0.88. The observed and simulated data are basically near the 1:1 line, and the slope of the trend line is 0.81 (P < 0.001). The R^2^ value of the random forest model based on 62 Rs ground samples in the Qinghai-Tibet Plateau is 0.83, the observed and simulated data are basically near the 1:1 line, and the slope of the trend line is 0.74 (P < 0.001). The results show that the random forest constructed by combining multiple-source data such as remote sensing and ground observation can effectively estimate the key carbon flux of the Qinghai-Tibet Plateau.

Table 1 compares the NPP simulation results of the four models on the Qinghai-Tibet Plateau. In order to fairly compare the simulation results of each model, we extracted the NPP values of each model based on the spatial location of the observation data. The NPP results of the Qinghai-Tibet Plateau simulated by the random forest model are superior to the other three models. The correlation coefficient between the NPP simulated by the random forest model and the observed data of the ground sample points is 0.42, while the correlation coefficient between the MODIS product, GLOPEM model and CASA model and the observed NPP is 0.38, 0.24 and 0.23, respectively. From the root mean square error, the results of random forest simulation improved 18.32% on average compared with the other three models. From the percentage of deviation and the average absolute percentage error, the random forest model is also more suitable for the Qinghai-Tibet Plateau than the other three models.

**Table 1:** Comparison of four NPP models

NPP	MODIS	GLOPEM	CASA	Mean	Random forest	Difference (%)
r	0.38	0.24	0.23	0.28	0.42	+ 49.00
RMSE	258.62	323.99	264.37	282.33	230.60	+ 18.32
PBIAS	− 28.09	− 54.62	− 35.33	− 39.35	− 25.74	+ 34.58
MAPE	88.61	105.08	81.18	91.62	68.41	+ 25.33

r is the correlation coefficient, RMSE is the root mean square error, PBIAS is the percentage of deviation, MAPE is the average absolute percentage error, Mean is the average value of NPP simulated by MODIS, GLOPEM and CASA models, and Difference is the percentage of difference between Random Forest and Average

**Fig. 3:**
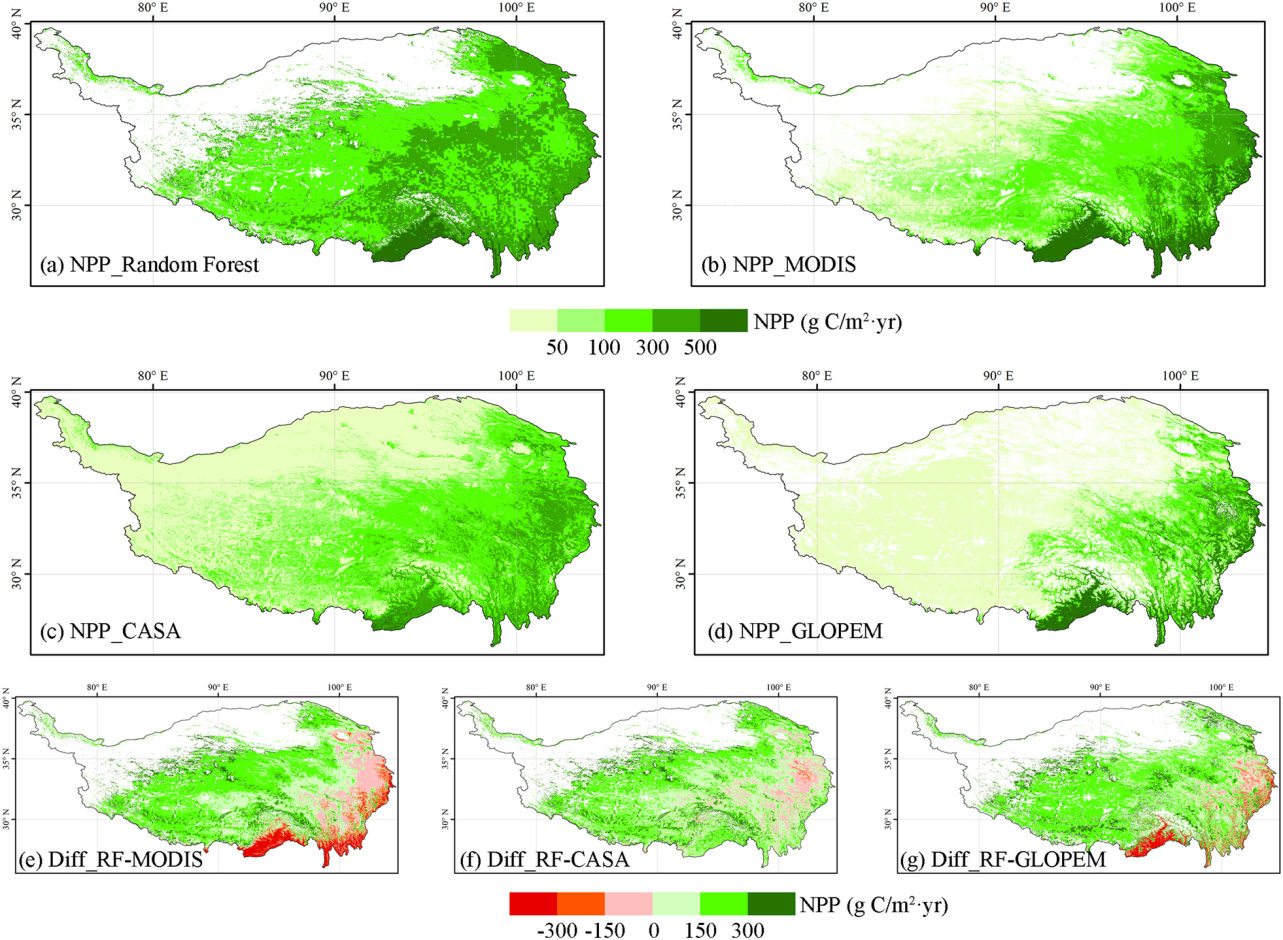
Four models simulate the spatial pattern of NPP and its differences. **a** NPP of random forest simulation; **b** NPP products of MODIS; **c** NPP simulated by CASA; **d** NPP simulated by GLOPEM; **e** NPP difference between random forest and MODIS; **f** NPP difference between random forest and CASA; **g** NPP difference between random forest and GLOEPM

By comparing the NPP spatial pattern of the Qinghai-Tibet Plateau simulated by different models, we find that MODIS NPP products, CASA models and GLOPEM have a large underestimation of the NPP of the Qinghai-Tibet Plateau (Fig. 3). The NPP results of random forest simulation are generally higher than 100 g C/m^2^ year, while the NPP results of the other three models are generally lower than 100 g C/m^2^ year in the vast central and western regions of the Qinghai-Tibet Plateau, especially the GLOPEM model is lower than 50 g C/m^2^ year. We also compared the spatial differences between the random forest and the other three models. The NPP of the random forest simulation is higher than the other three models in the central and western regions of the Qinghai-Tibet Plateau, while there are differences in a small part of the eastern regions with relatively high vegetation coverage. In general, the NPP products of MODIS, the NPP simulated by CASA model and GLOPEM model are underestimated to a large extent, which may seriously affect the accuracy of carbon sink estimation in the Qinghai-Tibet Plateau.

### Rs estimation of Qinghai-Tibet Plateau

Table 2 shows the simulation results of the two models for the Rs of the Qinghai-Tibet Plateau. In order to compare the simulation results of the two models fairly, we still extract the spatial results of the Rs of the two models based on the coordinates of the observed data. The results of Rs simulated by random forest model in Qinghai-Tibet Plateau are better than that of quantile regression forest model. The correlation coefficient between the Rs simulated by the random forest model and the ground sample observation data is 0.70, while the correlation coefficient between the quantile regression forest model and the observed Rs is 0.65. From the root mean square error, the results of random forest simulation improved 7.31% on average compared with the quantile regression forest model. From the percentage of deviation and the average absolute percentage error, the random forest model is also more suitable for the Qinghai-Tibet Plateau than the quantile regression forest model, but the difference is not as obvious as NPP.

**Table 2:** Comparison of two Rs models

Rs	Quantile regression forest model	Random forest	Difference (%)
r	0.65	0.70	7.31
RMSE	0.73	0.59	18.92
PBIAS	− 5.30	− 0.31	94.17
MAPE	15.98	10.96	31.43

r is the correlation coefficient, RMSE is the root mean square error, PBIAS is the percentage of deviation, MAPE is the average absolute percentage error, and Difference is the percentage of difference between Random Forest and Quantitative Regression Forest Model

**Fig. 4:**
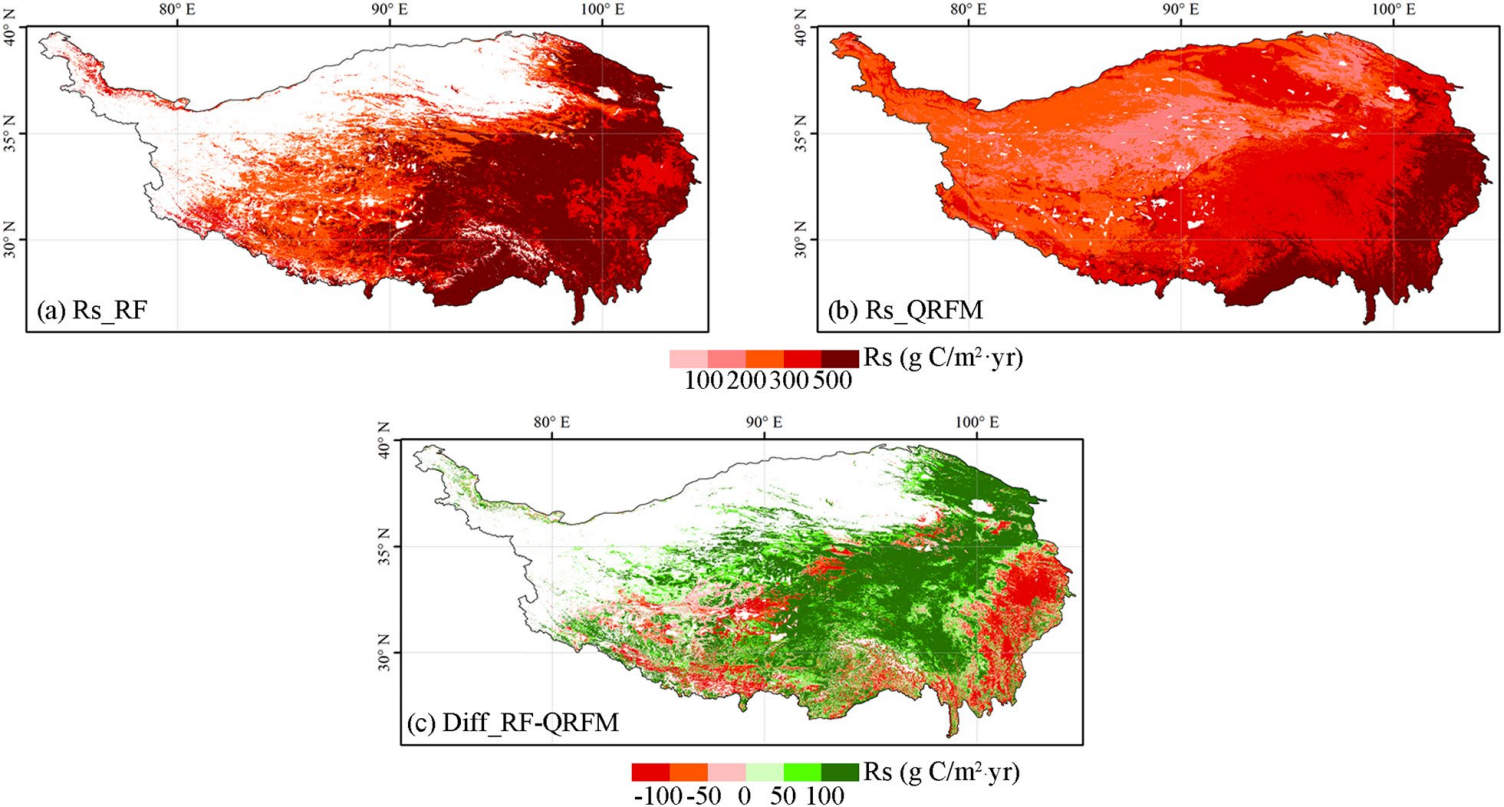
The two models simulate the spatial distribution pattern of Rs and their differences. **a** Rs simulated by random forest model; **b** Rs of QRFM; **c** Rs difference between random forest and QRFM

By comparing the spatial pattern of Rs in the Qinghai-Tibet Plateau simulated by the two models (Fig. 4), we find that the quantile regression forest model has a large underestimation of Rs in the Qinghai-Tibet Plateau. The Rs simulated by the random forest model have obvious east–west differentiation characteristics in the Qinghai-Tibet Plateau, and generally higher than 500 g C/m^2^ year in the east. The Rs simulated by quantile regression forest model also have east–west differentiation characteristics in the Qinghai-Tibet Plateau, but the difference is smaller than that of the random forest model. The Rs in most areas of the east are between 300 and 500 g C/m^2^ year. Comparing the spatial difference between the two models, it was found that the quantile regression forest models in most areas of the Qinghai-Tibet Plateau underestimated Rs.

### NEP estimation of Qinghai-Tibet Plateau

**Fig. 5:**
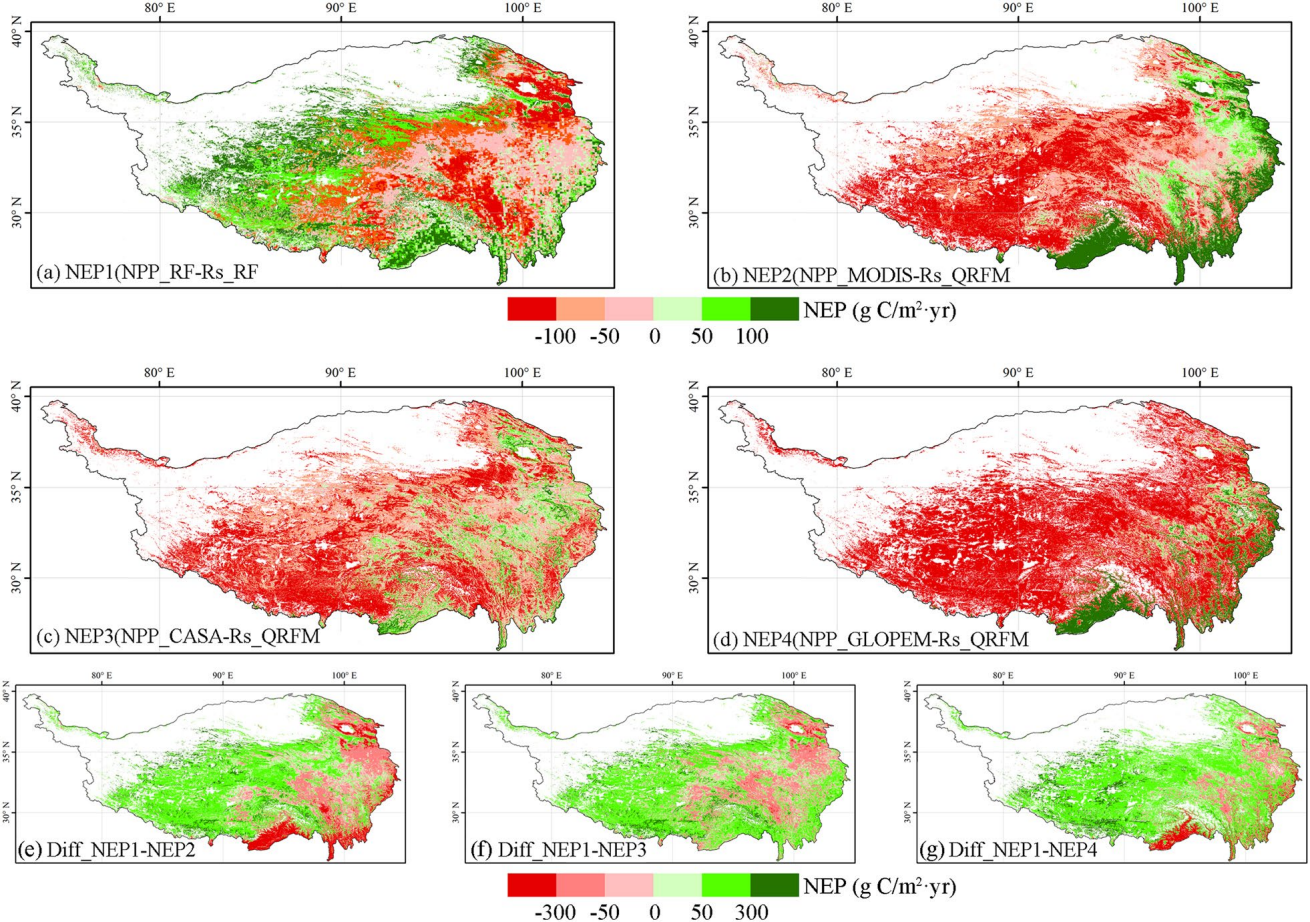
Various models simulate the spatial pattern of NEP and its differences. **a** NEP1, NPP simulated by random forest model minus Rh simulated by random forest and linear regression model; **b** NEP2, NPP of MODIS minus Rh simulated by QRFM and linear regression model; **c** NEP3, NPP simulated by CASA minus Rh simulated by QRFM and linear regression model; **d** NEP4, NPP simulated by GLOPEM minus Rh simulated by QRFM and linear regression model; **e** Difference between NEP1 and NEP2; **f** Difference between NEP1 and NEP3; **g** Difference between NEP1 and NEP4

Based on NPP and Rs simulated by various models, we compared the spatial pattern of carbon source and sink (NEP) in the Qinghai-Tibet Plateau estimated by four pairs of models and their differences (Fig. 5). The NEP simulated by the random forest model shows that the spatial pattern of carbon sinks in the Qinghai-Tibet Plateau presents a law of east–west differentiation, and the spatial heterogeneity is high. The west is generally a carbon sink area, while the east shows a carbon source. The other three pairs of model results show that the Qinghai-Tibet Plateau is a carbon source in general, and only the area with high vegetation coverage in the eastern part of the Qinghai-Tibet Plateau is a carbon sink. The results of spatial differences show that the carbon sink of the Qinghai-Tibet Plateau is underestimated to a large extent by the other three pairs of models except the random forest model. Most regions underestimate more than 50 g C/m^2^ year, which may lead to the misjudgment of whether the Qinghai-Tibet Plateau is a carbon sink or a carbon source.

**Fig. 6:**
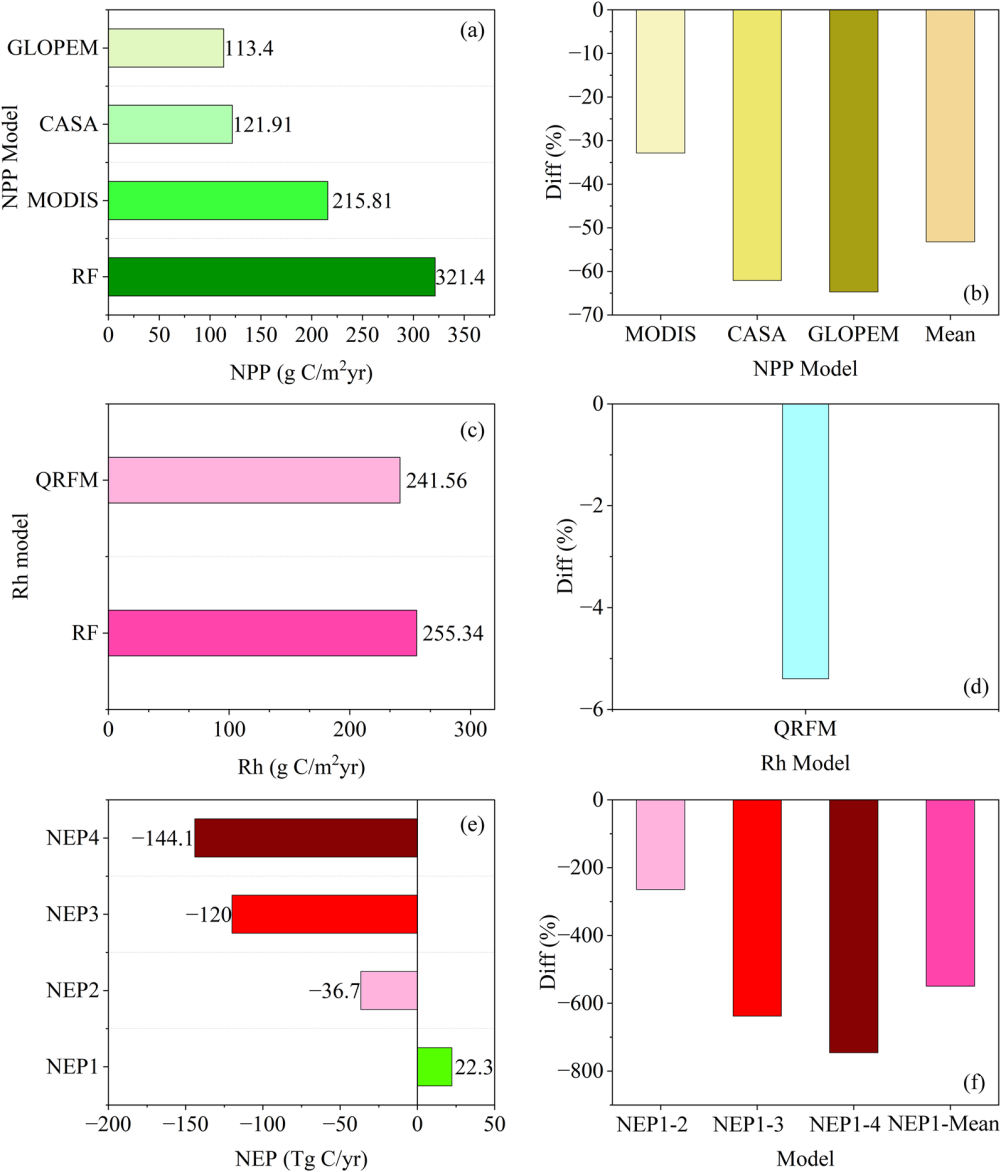
Various models simulate the carbon sink and its component differences in the Qinghai-Tibet Plateau. **a** NPP simulated by four models; **b** The percentage difference between the mean NPP of random forest simulation and that of other three models; **c** Rh simulated by random forest model and quantile regression forest model; **d** The percentage difference of Rh mean simulated by random forest model and quantile regression forest model; **e** NEP simulated by multiple combination models; **f** Percentage of difference between NEP1 and NEP2, NEP3, NEP4 and their mean values

Figure 6 shows that the difference in NPP estimates of the four models is greater than Rs, and there are differences in the amount and direction of carbon sink estimates of the Qinghai-Tibet Plateau. For NPP, the random forest model estimates an average of 321.4 g/Cm^2^ year, while other models underestimate it between 30 and 70%. For Rs, the average estimate of random forest model is about 255.3 g/Cm^2^ year, and the estimate of quantile regression forest model is relatively low, with an underestimation of about 5%. The random forest model estimates that the Qinghai-Tibet Plateau is a carbon sink in general, and will absorb 22.3 Tg of C per year (the average carbon sink intensity is 68.06 g/Cm^2^ year). The other three models believe that the Qinghai-Tibet Plateau is a carbon source area in general. The GLOPEM estimation results show that the Qinghai-Tibet Plateau may emit 144.1 Tg C (average carbon source intensity is 128.16 g/Cm^2^ year) per year, the CASA model estimates that the Qinghai-Tibet Plateau will emit about 120 Tg C (average carbon source intensity is 119.65 g/Cm^2^ year) per year, and the MODIS product estimates that the Qinghai-Tibet Plateau will emit about 36.7 Tg C (average carbon source intensity is 25.75 g/Cm^2^ year) per year. In general, the accuracy of carbon flux estimation of the Qinghai-Tibet Plateau by different models is quite different.

## Discussion

### Comparison of carbon source and sink simulation methods

The top-down atmospheric retrieval method can estimate the real-time changes of carbon sources and sinks worldwide [60], but its spatial resolution is low, and the retrieval accuracy is limited by the number and distribution of atmospheric observation stations, which cannot distinguish between man-made carbon emissions and natural process carbon emissions [61]. The bottom-up ecosystem process model method can simulate all aspects of the carbon cycle process, and has strong physical interpretation ability [62], but the structure and parameters of the model have great uncertainty [63]. The assumption of ecosystem model is usually based on carbon balance, while in the actual natural environment, the ecosystem is usually in a state of non-equilibrium disturbance, especially under the dual impact of human activities and climate change [64–66]. In addition, the widely used carbon source and sink estimation method based on carbon flux sites or ecological sample sites has high accuracy and can realize long-term continuous positioning observation of ecosystem carbon flux on a fine time scale [67, 68]. However, this method is greatly affected by the terrain and meteorological conditions, especially in the Qinghai-Tibet Plateau region. In the process of scaling up, there are problems such as insufficient sample representation, high cost and large spatial heterogeneity [39]. Satellite remote sensing products are increasingly used in terrestrial ecosystem carbon sink estimation because they can identify different underlying surface conditions, are suitable for large-scale carbon cycle monitoring, have strong real-time updating capability, high accuracy and spatial continuity, and have unparalleled cost-effectiveness [69–71]. However, there is usually a scale difference between the ecosystem carbon flux estimated by remote sensing data and the carbon flux estimated based on sample sites, and there is a systematic deviation between the estimates based on remote sensing products and the results estimated by sample sites [72]. Direct comparison of carbon input and output of ecosystems with scale differences has great uncertainty in the estimation of ecosystem carbon sink, which is one of the main reasons for the large difference in the estimation results of different methods [39, 72].

Figures 3 and 4 show that other models have different degrees of underestimation of NPP and Rs compared with random forest models. One reason is that most of the other models use remote sensing data as the main input source. Although remote sensing data can ensure spatial continuity, the ability to capture details of the Qinghai-Tibet Plateau, a region with complex terrain and sparse vegetation, is insufficient [32, 73, 74]. Therefore, remote sensing data in the Qinghai-Tibet Plateau is more suitable as a supplementary source of information than the main data [75]. Another important reason is that compared with the random forest model, these models lack the key ground sample observation information as a supplement. The Qinghai-Tibet Plateau has a complex terrain and is extremely vulnerable to the impact of climate change. In recent years, the intensity of human activities has gradually increased [12]. The traditional empirical model can no longer meet the requirements for the simulation and assessment of the carbon source and sink of the ecosystem in the region, and it is more necessary to supplement the observation information of the ground sample points to improve the reliability and applicability of the model [75]. Therefore, we propose a multiple scale information fusion method for carbon sink estimation that uses machine learning as a bridge to connect multiple-source data such as remote sensing and ground sample observation data. This method can not only achieve a wide range of carbon source and sink spatial simulation, but also ensure a high accuracy, so it is more suitable for the Qinghai-Tibet Plateau than other models. Machine learning can explore the nonlinear relationships between multi-source data, provide more accurate simulation results, and have stronger inclusiveness towards the data itself. It also has the characteristics of easy operation, economy, and applicability to multiple scales [76]. Therefore, compared to traditional carbon sink estimation methods, it is more suitable for carbon sink estimation in the Qinghai Tibet Plateau.

### Estimation difference of carbon sink in Qinghai-Tibet Plateau

The carbon sink estimates of the Qinghai-Tibet Plateau vary greatly among different models (Fig. 5). It is estimated that the Qinghai-Tibet Plateau is a carbon source or weak carbon sink through remote sensing products and simple linear models [77, 78]. The carbon source level of the Qinghai-Tibet Plateau is estimated to be 39.05 g/Cm^2^ year by using the model estimated by remote sensing products [32]. Due to the lack of ground sample data to optimize and constrain the model, these methods have produced a large deviation in the estimation of carbon sink results, especially in the Qinghai-Tibet Plateau, a region with complex terrain [79]. The study of combining the ground sample observation data with the model to constrain it shows that the Qinghai-Tibet Plateau is a carbon sink in general, with a carbon sink level of about 43.16 Tg C/year, and its carbon sink capacity may weaken in the future with the impact of climate change [36]. At the same time, we also emphasize that for the region with complex conditions such as the Qinghai-Tibet Plateau, more ground sample data are needed to improve the model of carbon sink estimation on the Qinghai-Tibet Plateau, otherwise the results may have a large deviation [80]. For example, only 46 observation data were used to estimate the terrestrial ecological carbon sink in China, and only 4 observations were made in the Qinghai-Tibet Plateau area, and the results obtained were more than 5 times higher than those of other scholars [39, 81].

Our results show that the Qinghai-Tibet Plateau is a carbon sink, about 22.3 Tg C/year (Fig. 6), which is also supported by relevant studies [30, 34, 82]. For example, a large number of studies based on field observations show that the carbon sink of China’s terrestrial ecosystem is 0.2–0.3 Pg C/year [1, 39, 68], and the Qinghai-Tibet Plateau is a carbon sink in general, accounting for about 10% of China's carbon sink [62, 83]. The main reason for the difference in the estimation of carbon sinks in the Qinghai-Tibet Plateau is the lack of sufficient ground sample observation data to optimize and constrain the model. The lack of observation data or a small amount of observation data may cause a large deviation in the estimation results [79, 80]. Second, the applicability of remote sensing data in the Qinghai-Tibet Plateau is insufficient. Due to the complex underlying surface conditions of the Qinghai-Tibet Plateau itself, the remote sensing data may not be able to completely retrieve the vegetation level of the Qinghai-Tibet Plateau, resulting in a serious underestimate of NPP [32, 84]. Therefore, the multiple scale information fusion carbon sink estimation method designed by us, which uses machine learning as a bridge to connect multiple-source data such as remote sensing and ground sample observation data, can not only preserve the spatial continuity of remote sensing data, capture spatial details, but also combine the ground sample observation data to optimize and constrain the model, making the simulation results more accurate.

### Impacts, limitations, and prospects

In this study, we designed a multiple scale information fusion method for carbon sink estimation that uses machine learning as a bridge to connect multiple-source data such as remote sensing and ground sample observation data. This method can not only preserve the spatial continuity of remote sensing data, capture spatial details, but also combine the ground sample observation data to optimize and constrain the model to obtain a set of spatially continuous, statistically reliable carbon input and output data, Make the simulation results more accurate. Then, based on the carbon input and output data of scale matching, the carbon sink level and its spatial pattern of the Qinghai-Tibet Plateau from 2000 to 2018 are estimated. In addition, we quantitatively evaluated the carbon sink results simulated by various models, and discussed the differences between the models. This study answers the controversial question of whether the Qinghai-Tibet Plateau is a carbon source or a carbon sink, and emphasizes the importance of carbon process simulation in areas with complex terrain and vulnerable to climate change, such as the Qinghai-Tibet Plateau, combined with ground sample observation data. The research results provide an important basis for accurately estimating the carbon sink and its spatial distribution in the Qinghai-Tibet Plateau, and provide a scientific reference for helping China achieve “carbon neutrality” and sustainable development by 2060.

This study uses multiple-source data, including climate-driven data sets, MODIS and related vegetation remote sensing products, and ground sample observations. Remote sensing products are affected by mixed pixels, and the fusion process of spatial data at different scales may affect the carbon sink estimation results [69]. In addition, although machine learning can explore nonlinear relationships between data, it has high requirements for the data itself, and abnormal data may have a significant impact on the results. Therefore, in the future, more and more representative samples are needed to further improve the reliability of the model [76]. Scaling the site data to the regional level places higher demands on the homogeneity of the samples, especially in the complex terrain of the Qinghai Tibet Plateau, where the uneven distribution of spatial sample points may lead to uncertainty in carbon si60nk estimation results in certain regions [41].

This study compares the key carbon fluxes of the Qinghai-Tibet Plateau simulated by various models, revealing that the Qinghai-Tibet Plateau is an important carbon sink. In the future, in-depth evaluation of the response mechanism of carbon sinks and related environmental factors (climate and land use change) may be a promising work [85, 86]. Especially in the context of global warming, global warming may cause the melting of permafrost in the Qinghai Tibet Plateau to release more soil carbon, while increasing water content may also promote vegetation photosynthesis, resulting in greater uncertainty in the estimation of carbon sources and sinks in the Qinghai Tibet Plateau region [87, 88]. In addition, it is necessary to combine the CMIP6 model to estimate carbon sink changes in different climate change and human activity scenarios on the Qinghai Tibet Plateau, especially to conduct research on the impact assessment of carbon sinks after a series of ecological projects, which can further provide basic support for related research on how ecosystems respond to climate change and has important practical significance [37, 73, 89].

## Conclusions

This study designed a multiple scale information fusion method for carbon sink estimation, which uses machine learning as a bridge to connect multiple-source data such as remote sensing and ground sample observation data. This method can not only preserve the spatial continuity of remote sensing data, but also combine the ground sample observation data to optimize and constrain the model, improve the simulation accuracy, so as to obtain a group of spatially continuous, statistically reliable carbon input and carbon output data. The results derived by the fusion of multiple scale data revealed that the Qinghai-Tibet Plateau, as an important ecological security barrier for China and even East Asia, has a carbon sink of about 22.3 Tg C/year. Compared with the random forest model, the traditional CASA, GLOPEM and MODIS products underestimated the carbon input of the Qinghai-Tibet Plateau by 30–70%, while the quantile regression forest model underestimated the carbon output of the Qinghai-Tibet Plateau by about 5%. These models underestimate carbon input more than carbon output, and may mistake the Qinghai-Tibet Plateau as a carbon source. This study provides an important basis for accurately estimating the carbon sink and its spatial distribution in the Qinghai-Tibet Plateau, and provides a scientific reference for helping China achieve “carbon neutrality” and sustainable development by 2060.

### Supplementary Information

**Additional file 1: Figure S1.** Scatter fitting of Rs and Rh based on observation data. **Figure S2.** Scatter fitting of NPP of random forest simulation and ground sample observation data. The green scatter points in the figure represent the training set of the random forest model, the red scatter points represent the verification set of the random forest model, the black dotted line represents the 1:1 line, and the blue color is realized as the fitting line. **Figure S3.** Scatter fitting of Ln (Rs) of random forest simulation and ground sample observation data. The green scatter points in the figure represent the training set of the random forest model, the red scatter points represent the verification set of the random forest model, the black dotted line represents the 1:1 line, and the blue line is the fitting line

**Additional file 2.** NPP/Rs/Rh ground observation sample information.

## Data Availability

The data that support the findings of this study were derived from the following resources available in the public domain: NPPMOSID dataset is available from https://e4ftl01.cr.usgs.gov/MOLT/MOD17A3HGF.006/. NPPCASA dataset can be downloaded from https://doi.org/10.6084/m9.figshare.20387889.v1. NPPGLO_PEM dataset can be downloaded from https://doi.org/10.6084/m9.figshare.20387874.v1. MOD12Q1 dataset can be downloaded from https://e4ftl01.cr.usgs.gov/MOTA/MCD12Q1.006/. Meteorological-driven dataset can be downloaded from https://doi.org/10.6084/m9.figshare.c.4557599.v1. The leaf area index is accessed from https://doi.org/10.6084/m9.figshare.20387928.v1. SRTMDEMUTM DEM is accessed from http://data.tpdc.ac.cn/en/data/acb49ce8-2bfe-4ab4-97ff-e6e727110703/. Global soil organic carbon dataset can be downloaded from https://soilgrids.org/. VHI can be downloaded from https://doi.org/10.6084/m9.figshare.19811854.v3. Soil respiration and Heterotrophic respiration dataset https://daac.ornl.gov/cgi-bin/dsviewer.pl?ds_id=1736.
