# Supplementary material for: The fusion of multiple scale data indicates that the carbon sink function of the Qinghai-Tibet Plateau is substantial
Source: Carbon Balance Manag. 2023 Sep 11;18:19. doi: 10.1186/s13021-023-00239-9 (PMC10494389; doi:10.1186/s13021-023-00239-9)
Supplement: Supplementary file 1 — Additional file 1: Figure S1. Scatter fitting of Rs and Rh based on observation data. Figure S2. Scatter fitting of NPP of random forest simulation and ground sample observation data. The green scatter points in the figure represent the training set of the random forest model, the red scatter points represent the verification set of the random forest model, the black dotted line represents the 1:1 line, and the blue color is realized as the fitting line. Figure S3. Scatter fitting of Ln (Rs) of random forest simulation and ground sample observation data. The green scatter points in the figure represent the training set of the random forest model, the red scatter points represent the verification set of the random forest model, the black dotted line represents the 1:1 line, and the blue line is the fitting line [file 13021_2023_239_MOESM1_ESM.docx]

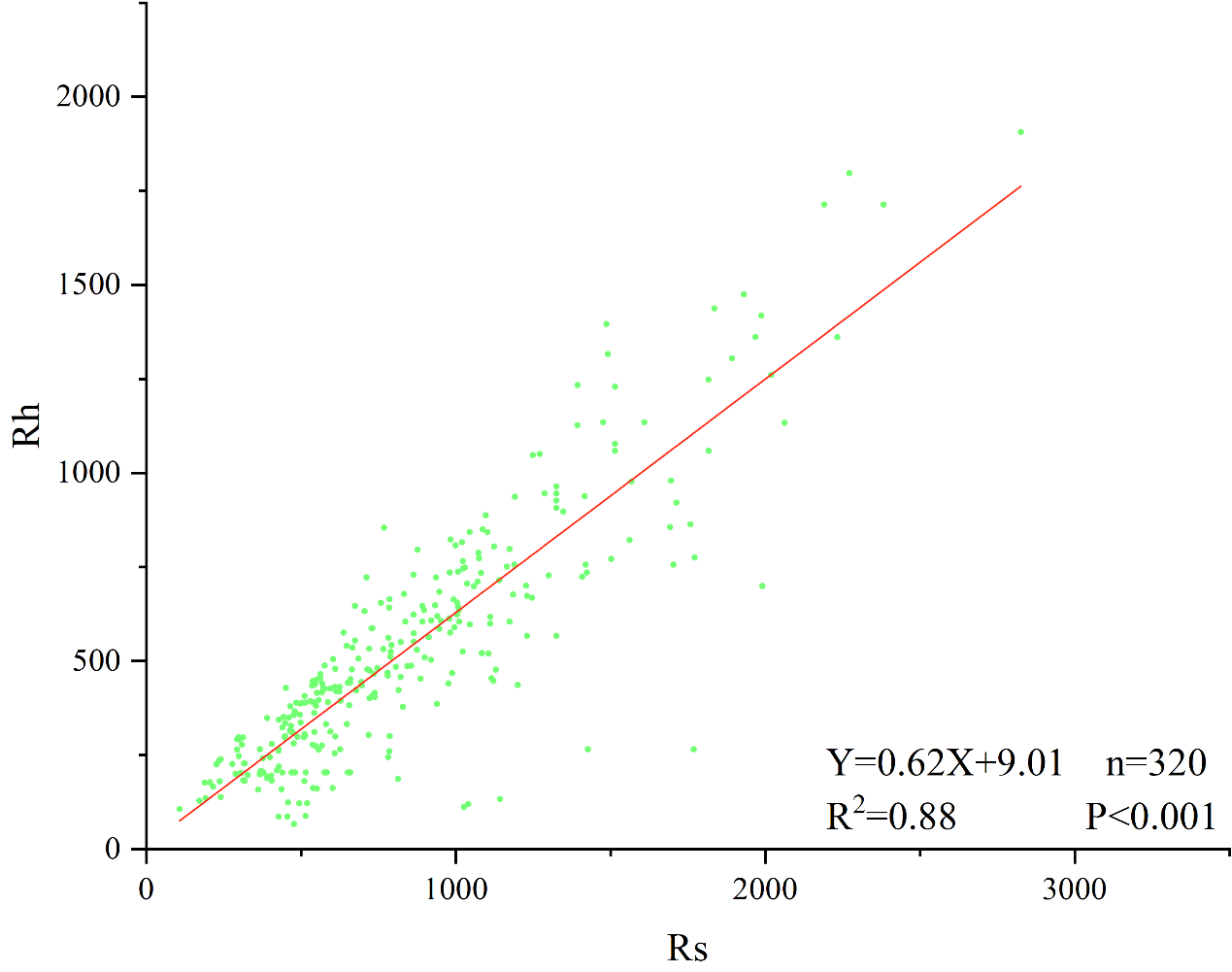


Figure S1 Scatter fitting of Rs and Rh based on observation data


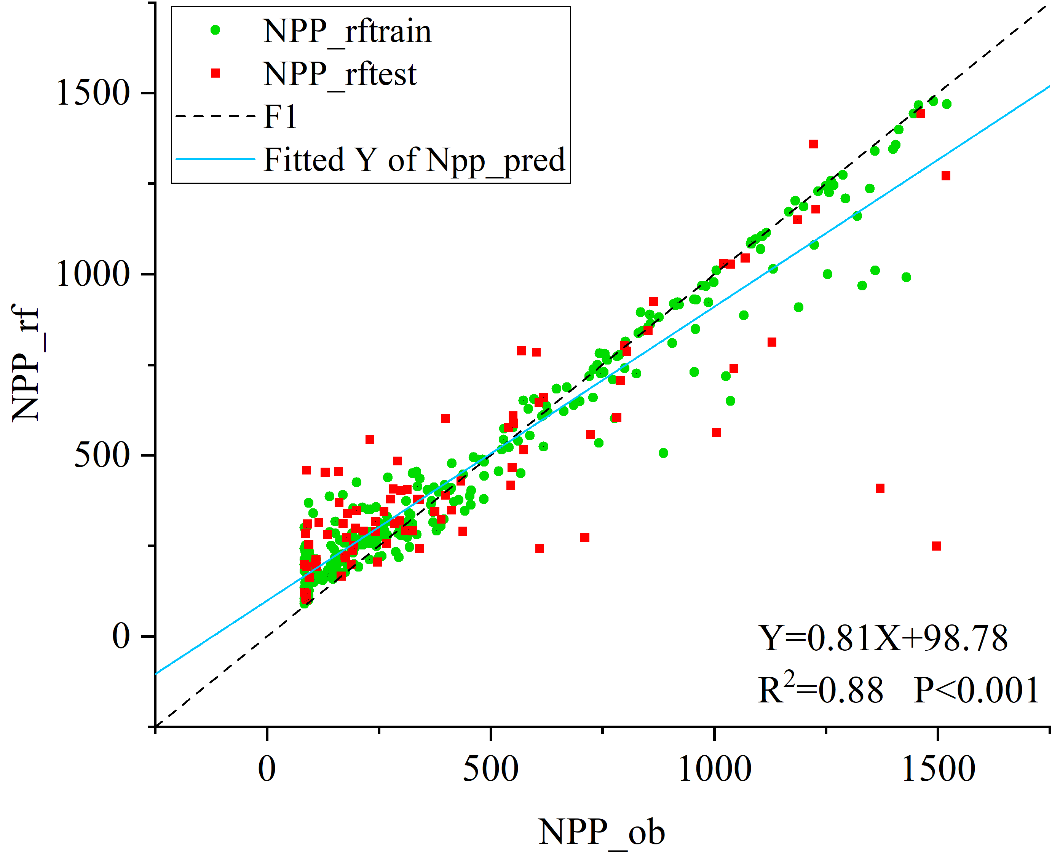


Figure S2 Scatter fitting of NPP of random forest simulation and ground sample observation data. The green scatter points in the figure represent the training set of the random forest model, the red scatter points represent the verification set of the random forest model, the black dotted line represents the 1:1 line, and the blue color is realized as the fitting line


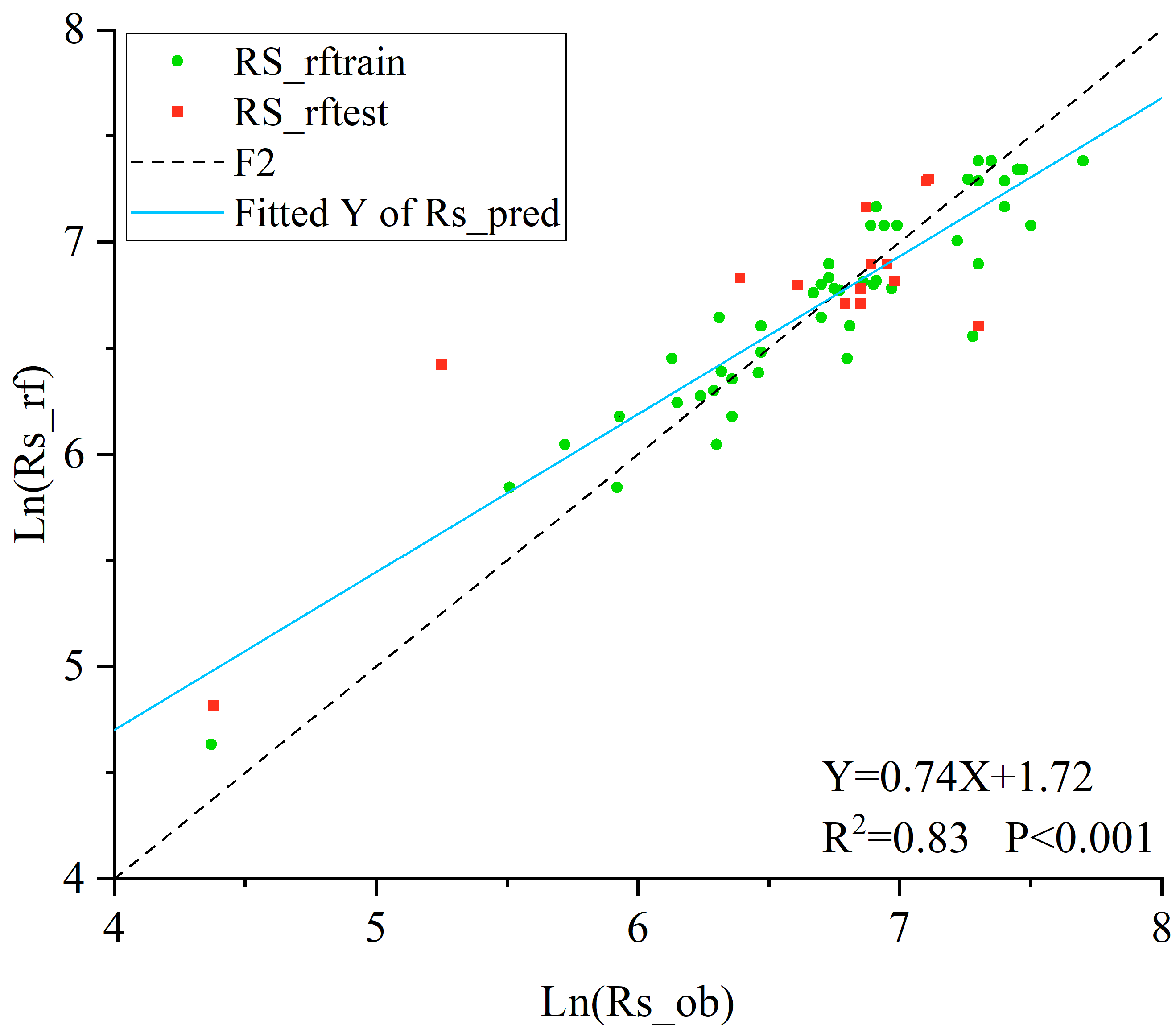


Figure S3 Scatter fitting of Ln (Rs) of random forest simulation and ground sample observation data. The green scatter points in the figure represent the training set of the random forest model, the red scatter points represent the verification set of the random forest model, the black dotted line represents the 1:1 line, and the blue line is the fitting line
